# Measurement properties of patient-reported outcome measures (PROMs) used in adult patients with chronic kidney disease: a systematic review protocol

**DOI:** 10.1136/bmjopen-2016-012014

**Published:** 2016-10-12

**Authors:** Olalekan Lee Aiyegbusi, Derek Kyte, Paul Cockwell, Tom Marshall, Thomas Keeley, Adrian Gheorghe, Melanie Calvert

**Affiliations:** 1 Centre for Patient Reported Outcomes Research, University of Birmingham, Birmingham, UK; 2 Institute of Applied Health Research, University of Birmingham, Birmingham, UK; 3 University Hospitals Birmingham NHS Foundation Trust, Queen Elizabeth Hospital Birmingham, Birmingham, UK; 4 Oxford Policy Management Ltd, Level 3 Clarendon Centre, Oxford, UK

**Keywords:** chronic kidney disease, measurement properties, psychometric property, quality of life, patient-reported outcome measures

## Abstract

**Introduction:**

Chronic kidney disease (CKD) is associated with symptoms that can significantly reduce the quality of life (QoL) of patients. Patient-reported outcome measures (PROMs) may facilitate the assessment of the impact of disease and treatment on the QoL, from a patient perspective. PROMs can be used in research and routine clinical practice.

**Methods and analysis:**

A systematic review of studies evaluating the measurement properties of PROMs in adults with CKD will be conducted. MEDLINE, EMBASE, PsycINFO and CINAHL Plus will be systematically searched from inception. Hand searching of reference lists and citations of included studies will be carried out. 2 reviewers will independently screen the titles and abstracts of all the studies retrieved during the systematic search to determine their eligibility. The COnsensus-based Standards for the selection of health Measurement Instruments (COSMIN) checklist will be used to appraise the methodological quality of the selected studies following the full-text review. Data on the study population, questionnaire characteristics and measurement properties will be extracted from the selected papers. Finally, a narrative synthesis of extracted data will be undertaken.

**Ethics and dissemination:**

Ethical permissions are not required for this study as data from published research articles will be used. Findings will be disseminated through publication in a peer-reviewed journal and presented at conferences. This systematic review will provide a comprehensive assessment of the measurement properties of PROMs currently available for use in adult patients with CKD and present evidence which may inform the selection of measures for use in research and clinical practice.

**Trial registration number:**

CRD42016035554.

**Strengths and limitations of this study**

- A key strength of this systematic review is that multiple databases will be searched by multiple independent reviewers, with no language or publication date restrictions, thus minimising the risk of selection bias.
- A further strength is that the methodological quality of selected studies will be evaluated using the validated COSMIN checklist.
- This review will include studies relating to chronic kidney disease in adults and exclude studies of all other kidney conditions and children, this decision has been taken to ensure the results are focused on the study research question.

## Introduction

Chronic kidney disease (CKD) is defined as the existence of kidney damage (ie, pathological abnormalities or markers of damage) for 3 months or more; and/or an estimated glomerular filtration rate (eGFR) <60 mL/min/1.73 m^2^ for 3 months or longer, with or without kidney damage.^1^

CKD affects up to 16% of adults in the UK^2^ and is associated with poor outcomes, with a high proportion of patients dying before reaching end-stage renal disease.^3^ ^4^ According to the UK Department of Health, the total cost of providing renal care by the National Health Service (NHS) was £1.64 billion in 2009–2010.^5^ Aside from these overt costs, the NHS bears other costs accrued from treating associated conditions while patients and caregivers have to contend with possible loss of income.^5^

Patients with CKD often suffer simultaneously from multiple symptoms related to the condition or side effects of their medical treatment.^6^ These clusters of symptoms may exert an adverse effect not only on their physical health but also on their psychological and emotional well-being; giving rise to what is described as a ‘symptom burden’.^7^ A review by Almutary *et al*^8^ identified 30 symptoms associated with CKD, with 5 symptoms (fatigue, drowsiness, pain, pruritus and dry skin) particularly common in all stages.^8^ They concluded that the overall symptom burden was high regardless of disease stage.^8^ This symptom burden is now acknowledged as the most important predictor of diminished health-related quality of life (HRQoL) in patients with CKD.^9^

In a clinical context, HRQoL refers to the manner in which the physical, emotional and social well-being of an individual is affected by a disease and/or its treatment.^10^ HRQoL can be measured using self-administered, validated questionnaires also known as patient-reported outcome measures (PROMs).^11^ ^12^

PROMs have numerous applications in clinical trials and routine clinical settings. They are employed in clinical trials as measures of effectiveness and pharmaceutical companies may use PROM data to support product approvals and labelling claims.^13^ PROMs can aid the reporting of serious adverse events due to drug toxicities^14^ and trial data can also influence clinical care and health policy.^15^ ^16^

In routine clinical practice, PROMs are mainly used as tools for benchmarking and hospital performance assessment.^17^ However, PROMs also have the potential to assist in the delivery of personalised care.^11^ ^17–21^

Currently, primary and secondary care records in the UK are being linked together such that patient information is more readily available to clinicians when required.^22^ The integration of patient-reported outcome (PRO) data with other routinely collected clinical data could provide an opportunity for revolutionising the UK healthcare system by facilitating the delivery of stratified medicine, enhancing clinical audits and assisting with the designing of pragmatic trials.^12^ ^21^

Given the vast array of PROMs in existence,^23^ the selection of an appropriate measure for any purpose requires the careful consideration of their measurement properties in order to derive any meaningful benefit from their application. It is crucial that such decisions are backed by the best evidence available, preferably from a recent systematic review.^12^

A scoping search identified two relevant systematic reviews namely: a 2010 review by Gibbons and Fitzpatrick and a 2012 review by Wyld *et al*.^24^ ^25^ Although both reviews presented evidence of the measurement properties of PROMs in CKD, there is a realistic possibility that more relevant research work has been performed since their publication.^26^ In addition, both studies had eligibility criteria which might have excluded potentially relevant studies.^24^ ^25^ Gibbons and Fitzpatrick restricted their review to studies published in English language and conducted in English-speaking populations within the UK, North America or Australasia. They also excluded studies with sample sizes <50.^24^ Wyld *et al*^25^ focused on studies that assessed preference-based (utility) measures excluding studies that evaluated HRQoL measures that did not have a utility component and only included studies conducted in other languages if they provided an English abstract.

Therefore, after considering the issues above, we concluded that a full systematic review is required. The aim of this systematic review will be to evaluate the measurement properties of PROMs currently available for use in adults with CKD.

This review will include studies that assess the measurement properties of generic, utility as well as disease-specific PROM instruments in patients at any stage of CKD. Recent methodological advances and guidelines^27–29^ will be employed to ensure that the most up-to-date and robust evidence is obtained.

## Methods and analysis

### Design

The protocol has been registered with PROSPERO (registration number CRD42016035554). It was developed using the Preferred Reporting Items for Systematic Review and Meta-Analysis Protocols (PRISMA-P) checklist.^29^

The review will be conducted and reported in compliance with the Preferred Reporting Items for Systematic Reviews and Meta-Analysis (PRISMA) guidelines.^30^

### Search strategy

The following electronic databases will be systematically searched from inception: MEDLINE (Ovid), EMBASE (Ovid), PsycINFO (Ovid) and CINAHL Plus (EBSCO). Literature search results will be uploaded to Endnote X7 (Thomson Reuters). There will be no publication period or language restrictions.^27^ The UK Renal Registry will be searched and expert recommendations from members of the review team will be followed up to help identify any additional measures currently under development.

The search strategy for MEDLINE was developed in consultation with an information specialist at the Institute of Applied Health Research, University of Birmingham. Two existing search filters, the sensitivity search filter developed by Terwee *et al*^31^ and the Oxford PROM filter,^32^ were combined with key terms for renal disease generated by the review team. The MEDLINE search strategy was adapted for use and tested on all the databases (see online supplementary appendix for the full search strategy).

10.1136/bmjopen-2016-012014.supp1 Supplementary appendix

### Selection of studies

To be considered for selection, an article must focus on PROMs used specifically for measuring QoL and/or CKD symptoms (the constructs of interest). In addition, the following inclusion and exclusion criteria will be applied.

#### Inclusion criteria

1. Articles reporting PROM development in all CKD populations.
2. Articles explicitly reporting the assessment of one or more psychometric properties for PROM(s) in all CKD populations.
3. Articles reporting cross-cultural validation of PROMs in all CKD populations.

#### Exclusion criteria

1. Clinician-assessed instruments.
2. Instrument development studies solely in patients with acute kidney injury.
3. Instruments developed solely for use in patients below 18 years of age.
4. Trials or studies evaluating the effectiveness of interventions where a PROM questionnaire is used as an end point.
5. Editorials, reviews and conference abstracts.

All titles and abstracts will be screened by two independent reviewers (OLA and TK/AG).

Full-text articles will be obtained for studies that potentially meet the eligibility criteria and will again be independently reviewed by the investigators (OLA and TK/AG).

Abstracts that do not provide the reviewers with enough information to make a decision will be taken forward for full-text screening, thus minimising the risk of missing potentially eligible articles.

Reasons for exclusion at the full-text stage of screening will be recorded. At any stage, if the reviewers are unable to reach a consensus, a third reviewer will be consulted (MC/DK). Hand searching of reference lists and citations of included papers will be conducted.

The process of selection will be summarised using a PRISMA flow diagram.

### Appraisal of the methodological quality of selected studies

All eligible papers will be independently appraised by two reviewers using the COnsensus-based Standards for the selection of health Measurement Instruments (COSMIN) checklist.^28^ ^33^ The COSMIN checklist is a validated critical appraisal tool designed for the systematic evaluation of the methodological quality of studies of the measurement properties of health measurement instruments.^28^

The COSMIN checklist employs a four-point rating scale which allows the rating of items relating to each measurement property as ‘excellent’, ‘good’, ‘fair’ or ‘poor’ depending on the methodological quality of each study. If a study meets all the requirements for an item, it is rated ‘excellent’ for that item. Conversely, if a study fails to meet the requirements for an item, it is given a lower rating commensurate to its quality.^28^

The overall quality rating for each measurement property is determined using the ‘worst score counts’ method.^28^ This means that the methodological quality for each measurement property will be determined by taking the lowest rating of its items.

The following measurement properties as defined by Mokkink *et al*^34^ will be evaluated:

1. Reliability—‘The proportion of the total variance in the measurements which is because of “true” differences among patients’.^34^
2. Internal consistency—‘The degree of the interrelatedness among the items’.^34^
3. Measurement error—‘The systematic and random error of a patient's score that is not attributed to true changes in the construct to be measured’.^34^
4. Content validity—‘The degree to which the content of an HR-PRO instrument is an adequate reflection of the construct to be measured’.^34^
5. Construct validity—‘The degree to which the scores of an HR-PRO instrument are consistent with hypotheses (for instance with regard to internal relationships, relationships to scores of other instruments, or differences between relevant groups) based on the assumption that the HR-PRO instrument validly measures the construct to be measured’.^34^
6. Cross-cultural validity—‘The degree to which the performance of the items on a translated or culturally adapted HR-PRO instrument are an adequate reflection of the performance of the items of the original version of the HR-PRO instrument’.^34^
7. Criterion validity—‘The degree to which the scores of an HR-PRO instrument are an adequate reflection of a “gold standard”’.^34^ The consensus by the COSMIN panel was that no gold standard exists for PROMs even though some authors consider widely used instruments as ‘gold standards’. An exception made by the panel is the comparison of a shortened measure to the original longer version, in which case, the original version can be regarded as the gold standard.^28^ ^33^
8. Responsiveness—‘The ability of an HR-PRO instrument to detect change over time in the construct to be measured’.^34^

### Data extraction

Data from selected studies will be extracted independently by two reviewers, using a data collection form, with disagreements resolved through discussion and, if necessary, consultation with a third reviewer. Extracted data will be presented in tables. Where appropriate, the results will be presented in separate sections/tables for instruments assessed in predialysis, dialysis and patients who had kidney transplant.

Data on the following will be extracted:

1. characteristics of the study population (including age, gender, ethnicity and stage of CKD);
2. questionnaire characteristics (including name/version, language, scoring method, domains, number of items);
3. evidence regarding the measurement properties of the questionnaire;
4. setting and purpose for which questionnaire is administered, interpretability and operational characteristics such as patient acceptability, and feasibility of administration for staff will also be reported.

### Data synthesis

Two sets of criteria will be used to assess the *quality of the measurement properties:*^27^ ^35^

- The quality criteria proposed by Terwee *et al*^35^ will be used to rate the *results of studies of measurement properties as* ‘positive’, ‘indeterminate’ or ‘negative’.^35^
- The modified criteria reported in Terwee 2011^27^ (originally proposed by the Cochrane Back Review Group)^36^ will be used to synthesis findings on the measurement property of each PROM across studies in order to ascertain the level of evidence for each instrument while taking into consideration the methodological quality of the selected studies. This overall level of evidence will be rated as ‘strong’, ‘moderate’, ‘limited’, ‘conflicting’ or ‘unknown’.^27^

## Dissemination

Findings will be disseminated through publication in a peer-reviewed journal and presented at conferences.

## Discussion

We acknowledge that studies will evaluate differing measurement properties; therefore, evidence might be limited or unavailable for some measurement properties.

This systematic review will present a comprehensive assessment of the measurement properties of PROMS currently available for use in adult patients with CKD and provide vital evidence which may inform the selection of measures for use in research and clinical practice.
