# Supplementary material for: Measurement properties of patient-reported outcome measures (PROMs) used in adult patients with chronic kidney disease: a systematic review protocol
Source: BMJ Open. 2016 Oct 12;6(10):e012014. doi: 10.1136/bmjopen-2016-012014 (PMC5073911; doi:10.1136/bmjopen-2016-012014)
Supplement: Supplementary appendix [file bmjopen-2016-012014supp_appendix.pdf]

MEDLINE (R) (Ovid) 1946 to 21/12/15

Note: Sets 1 to 6 are based on the Oxford PROM Filter.[1] Set 13 is the sensitive search filter for measurement properties developed by Terwee et al. [2]

1. (HR-PRO or HRPRO or HRQL or HRQoL or QL or QoL).ti,ab.

2. quality of life.mp.

3. (health index* or health indices or health profile*).ti,ab.

4. health status.mp.

5. ((patient or self or child or parent or carer or proxy) adj (appraisal* or appraised or report or reported or reporting or rated or rating or based or assessed or assessment*)).ti,ab.

6. ((disability or function or functional or functions or subjective or utility or utilities or wellbeing or well being) adj2 (index or indices or instrument or instruments or measure or measures or questionnaire* or profile or profiles or scale or scales or score or scores or status or survey or surveys)).ti,ab.

7. ((((patient adj reported adj outcome adj measure*) or patient) adj reported adj outcome*) or capability or capabilities).mp. [mp=title, abstract, original title, name of substance word, subject heading word, keyword heading word, protocol supplementary concept word, rare disease supplementary concept word, unique identifier]

8. 1 or 2 or 3 or 4 or 5 or 6 or 7

9. (Renal replacement therapy or APD or Automated Peritoneal Dialysis or CAPD, Continuous Ambulatory Peritoneal Dialysis or CCPD or Continuous cyclic peritoneal dialysis or dialysis or h*emofiltration or h*emodiafiltration or h*emodialysis or kidney transplant* or predialysis or renal replacement or renal transplant*).mp.

10. (CRF or chronic renal failure or CKF or chronic kidney failure or kidney disease* or renal disease or kidney failure or renal failure or CKD or chronic kidney disease or ESKD or end stage kidney disease or ESKF or end stage kidney failure or ESRF or end stage renal failure or ESRD or end stage renal disease or kidney insufficiency).mp.

11. Renal Insufficiency, Chronic/

12. 9 or 10 or 11

13. (((((((((((((((((((((((((((((Instrumentation or method* or Validation Studies or Comparative Study).mp. or psychometrics/ or psychometr*.mp. or clinimetr*.mp. or clinometr*.mp. or outcome assessment health care/ or outcome assessment*.ti,ab. or outcome measure*.mp. or observer variation/ or observer variation*.ti,ab. or Health Status Indicators/ or reproducibility of results/ or reproducib*.ti,ab. or discriminant analysis/ or reliab*.ti,ab. or unreliab*.ti,ab. or valid*.ti,ab. or coefficient of variation.ti,ab. or coefficient*.ti,ab. or homogeneity.ti,ab. or homogeneous.ti,ab. or internal consistency.ti,ab. or cronbach*.ti,ab.) and alpha*.ti,ab.) or item*.ti,ab.) and correlation*.ti,ab.) or selection*.ti,ab. or reduction*.ti,ab. or agreement.mp. or precision.mp. or imprecision.mp. or precise value*.mp. or test-retest.ti,ab. or test.ti,ab.) and retest.ti,ab.) or reliab*.ti,ab.) and test.ti,ab.) or retest.ti,ab. or stability.ti,ab. or interrater.ti,ab. or inter-rater.ti,ab. or intrarater.ti,ab. or intra-rater.ti,ab. or intertester.ti,ab. or inter-tester.ti,ab. or intratester.ti,ab. or intra-tester.ti,ab. or interobserver.ti,ab. or inter-observer.ti,ab. or intraobserver.ti,ab. or intra-observer.ti,ab. or intertechnician.ti,ab. or inter-technician.ti,ab. or intratechnician.ti,ab. or intra-technician.ti,ab. or interexaminer.ti,ab. or inter-examiner.ti,ab. or intraexaminer.ti,ab. or intra-examiner.ti,ab. or interassay.ti,ab. or inter-assay.ti,ab. or intraassay.ti,ab. or intra-assay.ti,ab. or interindividual.ti,ab. or inter-individual.ti,ab. or intraindividual.ti,ab. or intra-individual.ti,ab. or interparticipant.ti,ab. or inter-participant.ti,ab. or intraparticipant.ti,ab. or intra-participant.ti,ab. or kappa*.ti,ab. or kappa's.ti,ab. or repeatab*.mp. or replicab*.mp. or repeated.mp.) and measure*.mp.) or finding*.mp. or result*.mp. or test*.mp. or generaliza*.ti,ab. or generalisa*.ti,ab. or concordance.ti,ab. or intraclass.ti,ab.) and correlation*.ti,ab.) or discriminative.ti,ab. or known group.ti,ab. or factor analysis.ti,ab. or factor analyses.ti,ab. or factor structure.ti,ab. or factor structure.ti,ab. or dimension*.ti,ab. or subscale*.ti,ab. or multitrait.ti,ab.) and scaling.ti,ab. and analysis.ti,ab.) or analyses.ti,ab. or item discriminant.ti,ab. or interscale correlation*.ti,ab. or error.ti,ab. or errors.ti,ab. or individual variability.ti,ab. or interval variability.ti,ab. or rate variability.ti,ab. or variability.ti,ab.) and analysis.ti,ab.) or value*.ti,ab. or uncertainty.ti,ab.) and measurement.ti,ab.) or measuring.ti,ab. or standard error of measurement.ti,ab. or sensitiv*.ti,ab. or responsive*.ti,ab. or limit*.ti,ab.) and detection.ti,ab.) or minimal detectable concentration.ti,ab. or interpretab*.ti,ab. or minimal.ti,ab. or minimally.ti,ab. or clinical.ti,ab. or clinically.ti,ab.) and important.ti,ab.) or significant.ti,ab. or detectable.ti,ab.) and change.ti,ab.) or difference.ti,ab. or small*.ti,ab.) and real.ti,ab.) or detectable.ti,ab.) and change.ti,ab.) or difference.ti,ab. or meaningful change.ti,ab. or ceiling effect.ti,ab. or floor effect.ti,ab. or Item response model.ti,ab. or IRT.ti,ab. or Rasch.ti,ab. or Differential item functioning.ti,ab. or DIF.ti,ab. or computer adaptive testing.ti,ab. or item bank.ti,ab. or cross-cultural equivalence.ti,ab.

14. (PRO integration or Clinical PRO application* or telePRO or automated PRO algorithm* or screening purpose* or PRO questionnaire* or Patient-reported outcome questionnaire* or Patient-reported symptom* or Patient-centred care or Patient self-report* or Self-report health or Self-rated health or Self-reported measure* of health or Health outcome* or Health communication* or Hospital performance evaluation* or Automated telephone survey system* or paper-based survey* or web-based survey* or web-based PRO platform* or web-based system* or PRO collection* or PRO measure* or PRO intervention* or PRO assessment intervention* or PRO data or PRO assessment* or Routine PRO assessment* or Routine PRO collection or Symptom assessment* or Symptom monitoring or Symptom data or Functional status or Electronic PRO assessment* or Electronic PRO system* or ePRO or

ePRO* or ePRO system* or PRO system* or Generic PRO system* or PRO-based clinical alert system*).mp.

15. 13 or 14

16. 8 and 12 and 15

EMBASE (Ovid) 1974 to 21/12/15

Note: Set 4 is the sensitive search filter for measurement properties developed by Terwee et al. and adapted for EMBASE.[2]

1. (Renal replacement therapy or APD or Automated Peritoneal Dialysis or CAPD, Continuous Ambulatory Peritoneal Dialysis or CCPD or Continuous cyclic peritoneal dialysis or dialysis or h*emofiltration or h*emodiafiltration or h*emodialysis or kidney transplant* or predialysis or renal replacement or renal transplant*).mp.

2. (CRF or chronic renal failure or CKF or chronic kidney failure or kidney disease* or renal disease or kidney failure or renal failure or CKD or chronic kidney disease or ESKD or end stage kidney disease or ESKF or end stage kidney failure or ESRF or end stage renal failure or ESRD or end stage renal disease or kidney insufficiency).mp.

3. (PRO integration or Clinical PRO application* or telePRO or automated PRO algorithm* or screening purpose* or PRO questionnaire* or Patient-reported outcome questionnaire* or Patient-reported symptom* or Patient-centred care or Patient self-report* or Self-report health or Self-rated health or Self-reported measure* of health or Health outcome* or Health communication* or Hospital performance evaluation* or Automated telephone survey system* or paper-based survey* or web-based survey* or web-based PRO platform* or web-based system* or PRO collection* or PRO measure* or PRO intervention* or PRO assessment intervention* or PRO data or PRO assessment* or Routine PRO assessment* or Routine PRO collection or Symptom assessment* or Symptom monitoring or Symptom data or Functional status or Electronic PRO assessment* or Electronic PRO system* or ePRO or ePRO* or ePRO system* or PRO system* or Generic PRO system* or PRO-based clinical alert system*).mp.

4. ((exp questionnaire/ or exp named inventories/ or questionnaires/) and rating scales/) or exp psychometry/ or exp outcome assessment/ or exp pain assessment/ or exp disability/ or exp validity/ or exp reliability/

5. *patient/ or *outcome assessment/ or *questionnaire/ or *"quality of life"/ or *self report/

6. 3 or 4

7. 1 or 2

8. 5 and 6 and 7

PsycINFO (Ovid) 1967 to 21/12/15

Note: Sets 1 to 6 are based on the Oxford PROM Filter.[1] Set 11 is the sensitive search filter for measurement properties developed by Terwee et al.[2]

1. (HR-PRO or HRPRO or HRQL or HRQoL or QL or QoL).ti,ab.

2. quality of life.mp.

3. (health index* or health indices or health profile*).ti,ab.

4. health status.mp.

5. ((patient or self or child or parent or carer or proxy) adj (appraisal* or appraised or report or reported or reporting or rated or rating or based or assessed or assessment*)).ti,ab.

6. ((disability or function or functional or functions or subjective or utility or utilities or wellbeing or well being) adj2 (index or indices or instrument or instruments or measure or measures or questionnaire* or profile or profiles or scale or scales or score or scores or status or survey or surveys)).ti,ab.

7. ((((patient adj reported adj outcome adj measure*) or patient) adj reported adj outcome*) or capability or capabilities).mp. [mp=title, abstract, heading word, table of contents, key concepts, original title, tests & measures]

8. 1 or 2 or 3 or 4 or 5 or 6 or 7

9. (Renal replacement therapy or APD or Automated Peritoneal Dialysis or CAPD, Continuous Ambulatory Peritoneal Dialysis or CCPD or Continuous cyclic peritoneal dialysis or dialysis or h*emofiltration or h*emodiafiltration or h*emodialysis or kidney transplant* or predialysis or renal replacement or renal transplant*).mp.

10. (CRF or chronic renal failure or CKF or chronic kidney failure or kidney disease* or renal disease or kidney failure or renal failure or CKD or chronic kidney disease or ESKD or end stage kidney disease or ESKF or end stage kidney failure or ESRF or end stage renal failure or ESRD or end stage renal disease or kidney insufficiency).mp.

11. (((((((((((((((((((((((((((((Instrumentation or method* or Validation Studies or Comparative Study).mp. or psychometrics/ or psychometr*.mp. or clinimetr*.mp. or clinometr*.mp. or outcome assessment health care/ or outcome assessment*.ti,ab. or outcome measure*.mp. or observer variation/ or observer variation*.ti,ab. or Health Status Indicators/ or reproducibility of results/ or reproducib*.ti,ab. or

discriminant analysis/ or reliab*.ti,ab. or unreliab*.ti,ab. or valid*.ti,ab. or coefficient of variation.ti,ab. or coefficient*.ti,ab. or homogeneity.ti,ab. or homogeneous.ti,ab. or internal consistency.ti,ab. or cronbach*.ti,ab.) and alpha*.ti,ab.) or item*.ti,ab.) and correlation*.ti,ab.) or selection*.ti,ab. or reduction*.ti,ab. or agreement.mp. or precision.mp. or imprecision.mp. or precise value*.mp. or test-retest.ti,ab. or test.ti,ab.) and retest.ti,ab.) or reliab*.ti,ab.) and test.ti,ab.) or retest.ti,ab. or stability.ti,ab. or interrater.ti,ab. or inter-rater.ti,ab. or intrarater.ti,ab. or intra-rater.ti,ab. or intertester.ti,ab. or inter-tester.ti,ab. or intratester.ti,ab. or intra-tester.ti,ab. or interobserver.ti,ab. or inter-observer.ti,ab. or intraobserver.ti,ab. or intra-observer.ti,ab. or intertechnician.ti,ab. or inter-technician.ti,ab. or intratechnician.ti,ab. or intra-technician.ti,ab. or interexaminer.ti,ab. or inter-examiner.ti,ab. or intraexaminer.ti,ab. or intra-examiner.ti,ab. or interassay.ti,ab. or inter-assay.ti,ab. or intraassay.ti,ab. or intra-assay.ti,ab. or interindividual.ti,ab. or inter-individual.ti,ab. or intraindividual.ti,ab. or intra-individual.ti,ab. or interparticipant.ti,ab. or inter-participant.ti,ab. or intraparticipant.ti,ab. or intra-participant.ti,ab. or kappa*.ti,ab. or kappa's.ti,ab. or repeatab*.mp. or replicab*.mp. or repeated.mp.) and measure*.mp.) or finding*.mp. or result*.mp. or test*.mp. or generaliza*.ti,ab. or generalisa*.ti,ab. or concordance.ti,ab. or intraclass.ti,ab.) and correlation*.ti,ab.) or discriminative.ti,ab. or known group.ti,ab. or factor analysis.ti,ab. or factor analyses.ti,ab. or factor structure.ti,ab. or factor structure.ti,ab. or dimension*.ti,ab. or subscale*.ti,ab. or multitrait.ti,ab.) and scaling.ti,ab. and analysis.ti,ab.) or analyses.ti,ab. or item discriminant.ti,ab. or interscale correlation*.ti,ab. or error.ti,ab. or errors.ti,ab. or individual variability.ti,ab. or interval variability.ti,ab. or rate variability.ti,ab. or variability.ti,ab.) and analysis.ti,ab.) or value*.ti,ab. or uncertainty.ti,ab.) and measurement.ti,ab.) or measuring.ti,ab. or standard error of measurement.ti,ab. or sensitiv*.ti,ab. or responsive*.ti,ab. or limit*.ti,ab.) and detection.ti,ab.) or minimal detectable concentration.ti,ab. or interpretab*.ti,ab. or minimal.ti,ab. or minimally.ti,ab. or clinical.ti,ab. or clinically.ti,ab.) and important.ti,ab.) or significant.ti,ab. or detectable.ti,ab.) and change.ti,ab.) or difference.ti,ab. or small*.ti,ab.) and real.ti,ab.) or detectable.ti,ab.) and change.ti,ab.) or difference.ti,ab. or meaningful change.ti,ab. or ceiling effect.ti,ab. or floor effect.ti,ab. or Item response model.ti,ab. or IRT.ti,ab. or Rasch.ti,ab. or Differential item functioning.ti,ab. or DIF.ti,ab. or computer adaptive testing.ti,ab. or item bank.ti,ab. or cross-cultural equivalence.ti,ab.

12. (PRO integration or Clinical PRO application* or telePRO or automated PRO algorithm* or screening purpose* or PRO questionnaire* or Patient-reported outcome questionnaire* or Patient-reported symptom* or Patient-centred care or Patient self-report* or Self-report health or Self-rated health or Self-reported measure* of health or Health outcome* or Health communication* or Hospital performance evaluation* or Automated telephone survey system* or paper-based survey* or web-based survey* or web-based PRO platform* or web-based system* or PRO collection* or PRO measure* or PRO intervention* or PRO assessment intervention* or PRO data or PRO assessment* or Routine PRO assessment* or Routine PRO collection or Symptom assessment* or Symptom monitoring or Symptom data or Functional status or Electronic PRO assessment* or Electronic PRO system* or ePRO or ePRO* or ePRO system* or PRO system* or Generic PRO system* or PRO-based clinical alert system*).mp.

13. 11 or 12

14. *Organ Transplantation/ or *Hemodialysis/ or *Kidneys/ or *Kidney Diseases/ or Renal Insufficiency, Chronic.mp.

15. 9 or 10 or 14

16. 8 and 13 and 15

CINAHL (via EBSCO host) inception to 21/12/15

Note: S1 is based on the Oxford PROM Filter.[1] S3 is the sensitive search filter for measurement properties developed by Terwee et al. [2]

S1. ((HR-PRO or HRPRO or HRQL or HRQoL or QL or QoL).ti,ab.) OR quality of life.mp. OR ((health index* or health indices or health profile*).ti,ab.) OR health status.mp. OR (((patient or self or child or parent or carer or proxy) adj (appraisal* or appraised or report or reported or reporting or rated or rating or based or assessed or assessment*)).ti,ab.) OR (((disability or function or functional or functions or subjective or utility or utilities or wellbeing or well being) adj2 (index or indices or instrument or instruments or measure or measures or questionnaire* or profile or profiles or scale or scales or score or scores or status or survey or surveys)).ti,ab.) OR (((((patient adj reported adj outcome adj measure*) or patient) adj reported adj outcome*) or capability or capabilities).mp)

S2. (MH "Kidney Failure, Chronic/TH/TM/TD/SU/SS/RF/RH/PF/PR/PC/PP/PA/NU/MO/ME/ET/EI/EP/ED/EC/DT/DI/CO/CL") OR "((Renal replacement therapy or APD or Automated Peritoneal Dialysis or CAPD, Continuous Ambulatory Peritoneal Dialysis or CCPD or Continuous cyclic peritoneal dialysis or dialysis or h*emofiltration or h*emodiafiltration or h*emodialysis or kidney transplant* or predialysis or renal replacement or renal transplant*).mp.) OR ((CRF or chronic renal failure or CKF or chronic kidney failure or kidney disease* or renal disease or kidney failure or renal failure or CKD or chronic kidney disease or ESKD or end stage kidney disease or ESKF or end stage kidney failure or ESRF or end stage renal failure or ESRD or end stage renal disease or kidney insufficiency).mp.) OR (*Organ Transplantation/ or *Hemodialysis/ or *Kidneys/ or *Kidney Diseases/ or Renal Insufficiency, Chronic.mp.)"

S3. ""((((((((((((((((((((((((((((((Instrumentation or method* or Validation Studies or Comparative Study).mp. or psychometrics/ or psychometr*.mp. or clinimetr*.mp. or clinometr*.mp. or outcome assessment health care/ or outcome assessment*.ti,ab. or outcome measure*.mp. or observer variation/ or observer variation*.ti,ab. or Health Status Indicators/ or reproducibility of results/ or reproducib*.ti,ab. or discriminant analysis/ or reliab*.ti,ab. or unreliab*.ti,ab. or valid*.ti,ab. or coefficient of variation.ti,ab. or coefficient*.ti,ab. or homogeneity.ti,ab. or homogeneous.ti,ab. or internal consistency.ti,ab. or cronbach*.ti,ab.) and alpha*.ti,ab.) or item*.ti,ab.) and correlation*.ti,ab.) or selection*.ti,ab. or reduction*.ti,ab. or agreement.mp. or precision.mp. or imprecision.mp. or precise value*.mp. or test-retest.ti,ab. or test.ti,ab.) and retest.ti,ab.) or reliab*.ti,ab.) and test.ti,ab.) or retest.ti,ab. or stability.ti,ab. or interrater.ti,ab. or inter-rater.ti,ab. or intrarater.ti,ab. or intra-rater.ti,ab. or intertester.ti,ab. or inter-tester.ti,ab. or intratester.ti,ab. or intra-tester.ti,ab. or interobserver.ti,ab. or inter-observer.ti,ab. or intraobserver.ti,ab. or intra-

observer.ti,ab. or intertechnician.ti,ab. or inter-technician.ti,ab. or intratechnician.ti,ab. or intra-technician.ti,ab. or interexaminer.ti,ab. or inter-examiner.ti,ab. or intraexaminer.ti,ab. or intra-examiner.ti,ab. or interassay.ti,ab. or inter-assay.ti,ab. or intraassay.ti,ab. or intra-assay.ti,ab. or interindividual.ti,ab. or inter-individual.ti,ab. or intraindividual.ti,ab. or intra-individual.ti,ab. or interparticipant.ti,ab. or inter-participant.ti,ab. or intraparticipant.ti,ab. or intra-participant.ti,ab. or kappa*.ti,ab. or kappa's.ti,ab. or repeatab*.mp. or replicab*.mp. or repeated.mp.) and measure*.mp.) or finding*.mp. or result*.mp. or test*.mp. or generaliza*.ti,ab. or generalisa*.ti,ab. or concordance.ti,ab. or intraclass.ti,ab.) and correlation*.ti,ab.) or discriminative.ti,ab. or known group.ti,ab. or factor analysis.ti,ab. or factor analyses.ti,ab. or factor structure.ti,ab. or factor structure.ti,ab. or dimension*.ti,ab. or subscale*.ti,ab. or multitrait.ti,ab.) and scaling.ti,ab. and analysis.ti,ab.) or analyses.ti,ab. or item discriminant.ti,ab. or interscale correlation*.ti,ab. or error.ti,ab. or errors.ti,ab. or individual variability.ti,ab. or interval variability.ti,ab. or rate variability.ti,ab. or variability.ti,ab.) and analysis.ti,ab.) or value*.ti,ab. or uncertainty.ti,ab.) and measurement.ti,ab.) or measuring.ti,ab. or standard error of measurement.ti,ab. or sensitiv*.ti,ab. or responsive*.ti,ab. or limit*.ti,ab.) and detection.ti,ab.) or minimal detectable concentration.ti,ab. or interpretab*.ti,ab. or minimal.ti,ab. or minimally.ti,ab. or clinical.ti,ab. or clinically.ti,ab.) and important.ti,ab.) or significant.ti,ab. or detectable.ti,ab.) and change.ti,ab.) or difference.ti,ab. or small*.ti,ab.) and real.ti,ab.) or detectable.ti,ab.) and change.ti,ab.) or difference.ti,ab. or meaningful change.ti,ab. or ceiling effect.ti,ab. or floor effect.ti,ab. or Item response model.ti,ab. or IRT.ti,ab. or Rasch.ti,ab. or Differential item functioning.ti,ab. or DIF.ti,ab. or computer adaptive testing.ti,ab. or item bank.ti,ab. or cross-cultural equivalence.ti,ab.)

S4. ((PRO integration or Clinical PRO application* or telePRO or automated PRO algorithm* or screening purpose* or PRO questionnaire* or Patient-reported outcome questionnaire* or Patient-reported symptom* or Patient-centred care or Patient self-report* or Self-report health or Self-rated health or Self-reported measure* of health or Health outcome* or Health communication* or Hospital performance evaluation* or Automated telephone survey system* or paper-based survey* or web-based survey* or web-based PRO platform* or web-based system* or PRO collection* or PRO measure* or PRO intervention* or PRO assessment intervention* or PRO data or PRO assessment* or Routine PRO assessment* or Routine PRO collection or Symptom assessment* or Symptom monitoring or Symptom data or Functional status or Electronic PRO assessment* or Electronic PRO system* or ePRO or ePRO* or ePRO system* or PRO system* or Generic PRO system* or PRO-based clinical alert system*).mp.)""

S5. S3 or S4

S6. S1 and S2 and S5

References

(1) PROM Group, A.M., Carolina Casañas i Comabella, Monica Hadi, Elizabeth Gibbons, Ray Fitzpatrick, Nia Roberts, *PROM GROUP CONSTRUCT & INSTRUMENT TYPE FILTERS.* 2010.

(2) Terwee, C.B., et al., *Development of a methodological PubMed search filter for finding studies on measurement properties of measurement instruments.* Qual Life Res, 2009. **18**(8): p. 1115-23.
